# Supplementary material for: The impact of comorbidity burden on outcomes following endovascular thrombectomy for acute ischemic stroke: A nationwide prospective observational study
Source: Eur Stroke J. 2025 Apr 12:23969873251332136. Online ahead of print. doi: 10.1177/23969873251332136 (PMC11993546; doi:10.1177/23969873251332136)
Supplement: sj-docx-1-eso-10.1177_23969873251332136 – Supplemental material for The impact of comorbidity burden on outcomes following endovascular thrombectomy for acute ischemic stroke: A nationwide prospective observational study [file sj-docx-1-eso-10.1177_23969873251332136.docx]

**Supplements**

***Supplementary Figure 1.***

EVT re-treatments**, n = 82**

Pre-stroke dependency status unknown, **n = 43**

Patients registered in Riksstroke and EVAS 2015**–**2021, **n = 5,291**

No EVT due to spontaneous reperfusion, **n = 90**

Included in the study, **n = 4,735**

Pre-stroke dependent (mRS 3-5), **n = 341**

Supplementary Figure 1: Flow chart describing the study population (n = 4,735).
EVT = EndoVascular Thrombectomy, mRS = modified Rankin Scale.

***Supplementary Table 1.*** Diagnoses included in the original Charlson Comorbidity Index and the weight of the individual diagnoses in the index.

| Diagnoses in the Charlson Comorbidity Index and ICD-10 code | Weight in the index |
| --- | --- |
| Solid tumor, non-metastatic (C00-76) | 2 |
| Solid tumor, metastatic (C77-79) | 6 |
| Leukemia/myeloma (C88-96) | 2 |
| Chronic liver disease, mild (B18, K70, K72) | 1 |
| Chronic liver disease, moderate/severe (K73-74) | 3 |
| Chronic kidney failure (N18) | 2 |
| COPD* (J44) | 1 |
| Rheumatoid arthritis, autoinflammatory syndrome (M04-05) | 1 |
| Peripheral vascular disease (I70, I73) | 1 |
| Congestive heart failure (I25, I50) | 1 |
| Myocardial infarction (I21-22) | 1 |
| Diabetes (E10) | 2 |
| Dementia (F00-03) | 1 |
| HIV/AIDS (B20-24) | 6 |
| Ulcer disease (K25-26) | 1 |
| Cerebrovascular disease (I67.9) | 1 |
| Hemiplegia (G83.1-3) | 1 |
| Lymphoma (C.81-86) | 2 |

***Supplementary Table 2.*** Peri- and postoperative complications for all EVT patients, and for recanalized and non-recanalized patients within the CCI groups.

| **Peri- and postoperative complications** | | | | | |
| --- | --- | --- | --- | --- | --- |
|  | **All** | **CCI 0** | **CCI 1** | **CCI 2** | **CCI ≥3** |
| **Perioperative complications** | 378 (8) | 136 (7.1) | 62 (8.9) | 96 (9.8) | 84 (7.3) |
| Recanalized | 283 (7.2) | 104 (6.5) | 50 (8.3) | 68 (8.3) | 61 (6.5) |
| Non-recanalized | 95 (12.2) | 32 (10.2) | 12 (12) | 28 (17.9) | 23 (11.2) |
| **Perforation/extravasation** | 69 (1.5) | 22 (1.1) | 8 (1.1) | 21 (2.2) | 18 (1.6) |
| Recanalized | 36 (0.9) | 13 (0.8) | 5 (0.8) | 11 (1.3) | 7 (0.7) |
| Non-recanalized | 33 (4.2) | 9 (2.9) | 3 (3) | 10 (6.4) | 11 (5.3) |
| **Other perioperative complication** | 309 (6.5) | 114 (6) | 54 (7.7) | 75 (7.7) | 66 (5.8) |
| Recanalized | 247 (6.2) | 91 (5.7) | 45 (7.5) | 57 (7) | 54 (5.7) |
| Non-recanalized | 62 (8) | 23 (7.3) | 9 (9) | 18 (11.5) | 12 (5.8) |
| **Postoperative complications** | 858 (18.1) | 300 (15.7) | 122 (17.5) | 202 (20.7) | 234 (20.4) |
| Recanalized | 667 (16.9) | 231 (14.4) | 92 (15.4) | 161 (19.7) | 183 (19.4) |
| Non-recanalized | 191 (24.6) | 69 (21.9) | 30 (30) | 41 (26.3) | 51 (24.8) |
| **Any ICH within 24 hours** | 1,038 (21.9) | 399 (20.8) | 145 (20.7) | 222 (22.8) | 272 (23.7) |
| Recanalized | 850 (21.5) | 325 (20.3) | 114 (19.0) | 180 (22.0) | 231 (24.5) |
| Non-recanalized | 188 (24.2) | 74 (23.5) | 31 (31) | 42 (26.9) | 41 (19.9) |
| **sICH** | 236 (5.0) | 77 (4.0) | 39 (5.6) | 57 (5.8) | 63 (5.5) |
| Recanalized | 177 (4.5) | 54 (3.4) | 28 (4.7) | 46 (5.6) | 49 (5.2) |
| Non-recanalized | 59 (7.6) | 23 (7.3) | 11 (11) | 11 (7.1) | 14 (6.8) |
| **Malignant infarction** | 113 (2.4) | 62 (3.2) | 10 (1.4) | 24 (2.5) | 17 (1.5) |
| Recanalized | 78 (2.0) | 44 (2.8) | 7 (1.2) | 15 (1.8) | 12 (1.3) |
| Non-recanalized | 35 (4.5) | 18 (5.7) | 3 (3.0) | 9 (5.8) | 5 (2.4) |
| **Severe infection** | 355 (7.5) | 126 (6.69) | 46 (6.6) | 92 (9.4) | 91 (7.9) |
| Recanalized | 272 (6.9) | 92 (5.8) | 38 (6.3) | 73 (8.9) | 69 (7.3) |
| Non-recanalized | 83 (10.7) | 34 (10.8) | 8 (8) | 19 (12.2) | 22 (10.7) |
| **Cardiovascular event** | 205 (4.3) | 56 (2.9) | 34 (4.9) | 44 (4.5) | 71 (6.2) |
| Recanalized | 166 (4.2) | 46 (2.9) | 26 (4.3) | 34 (4.2) | 60 (6.4) |
| Non-recanalized | 39 (5) | 10 (3.2) | 8 (8) | 10 (6.4) | 11 (5.3) |
| **Other severe complication*** | 126 (2.7) | 33 (1.7) | 17 (2.4) | 36 (3.7) | 40 (3.5) |
| Recanalized | 103 (2.6) | 27 (1.7) | 14 (2.3) | 30 (3.7) | 32 (3.4) |
| Non-recanalized | 23 (3.0) | 6 (1.9) | 3 (3.0) | 6 (3.8) | 8 (3.9) |
| Presented as numbers (%). *Complication without relation to EVT, for example severe fall accident CCI = Charlson Comorbidity Index, ICH = IntraCranial Hemorrhage, sICH = symptomatic IntraCranial Hemorrhage. | | | | | |
